# Preparation and Preliminary Dialysis Performance Research of Polyvinylidene Fluoride Hollow Fiber Membranes

**DOI:** 10.3390/membranes5010120

**Published:** 2015-03-19

**Authors:** Qinglei Zhang, Xiaolong Lu, Juanjuan Liu, Lihua Zhao

**Affiliations:** 1 Institute of Biological and Chemical Engineering, Tianjin Polytechnic University, Tianjin 300387, China; E-Mails: haiyang19802005@163.com (Q.Z.); 13516211811@139.com (L.Z.); 2 State Key Laboratory of Hollow Fiber Membrane Materials and Membrane Processes, Tianjin Polytechnic University, Tianjin 300387, China; 3 Tianjin Third Central Hospitals, Tianjin 300170, China; E-Mail: athena196121@163.com

**Keywords:** Polyvinylidene fluoride (PVDF), preparation, separation properties, high flux, dialysis performance

## Abstract

In this study, the separation properties of Polyvinylidene fluoride (PVDF) hollow fiber hemodialysis membranes were improved by optimizing membrane morphology and structure. The results showed that the PVDF membrane had better mechanical and separation properties than Fresenius Polysulfone High-Flux (F60S) membrane. The PVDF membrane tensile stress at break, tensile elongation and bursting pressure were 11.3 MPa, 395% and 0.625 MPa, respectively. Ultrafiltration (UF) flux of pure water reached 108.2 L∙h^−1^∙m^−2^ and rejection of Albumin from bovine serum was 82.3%. The PVDF dialyzers were prepared by centrifugal casting. The influences of membrane area and simulate fluid flow rate on dialysis performance were investigated. The results showed that the clearance rate of urea and Lysozyme (LZM) were improved with increasing membrane area and fluid flow rate while the rejection of albumin from bovine serum (BSA) had little influence. The high-flux PVDF dialyzer UF coefficient reached 62.6 mL/h/mmHg. The PVDF dialyzer with membrane area 0.69 m^2^ has the highest clearance rate to LZM and urea. The clearance rate of LZM was 66.8% and urea was 87.7%.

## 1. Introduction

Hemodialysis (HD) is a relatively safe purification technique for curing renal failure. The core element is Ultrafiltration hollow fiber membrane (HFM) [1,2]. The core aim for hemodialysis is to remove “middle” and “small” molecules toxin, such as β_2_-MG and urea nitrogen. There are more and more polymeric materials in HD to improve the clearance for small molecules and blood compatibility. Nowadays, polyethersulfone (PES) and polysulfone (PSF) hemodialysis membranes show better biocompatibility, functional effectiveness, and small substances clearance than other membranes; so they are widely used in hemodialysis [3,4,5,6,7]. However, the clearance for “middle” molecules toxin and Ultra filtration (UF) flux of pure water are not ideal. At the same time, the membranes are prone to rupture in the dialysis process.

Great attention has been paid to Polyvinylidene fluoride (PVDF) by more and more people in the world for its outstanding properties. That can be explained by its high mechanical properties, thermal stability, and surface smoothness compared with other polymeric materials. Just for its outstanding properties, PVDF membranes have been extensively used in ultrafiltration/microfiltration and Membrane Bioreactor (MBR) separation technology. In addition, PVDF membranes are also widely applied in membrane distillation, membrane extraction, gas separation, and biomedical materials [8,9,10,11,12]. PVDF has gained worldwide attention in biomedical research owing to its excellent properties. Bouaziz A. considered PVDF as one of the artificial vascular materials [13]. 

Polyethylene glycol (PEG) has been extensively applied in the process of membrane preparation as a common additive. That can be attributed to its unique properties, such as non-irritating, good solubility, and fine compatibility [14]. Because of its good biocompatibility, it is widely used in biomedical materials. At the same time, there are many studies on the modification of membrane surface with PEG [15,16] or by blending PEG graft polymer during membrane preparation [17,18].

We have done some studies on the preparation of PVDF hollow fiber hemodialysis membranes. From the results, it can be seen that the mechanical performance and albumin from bovine serum (BSA) adsorption of PVDF membranes were better while separation properties were worse than Fresenius Polysulfone High-Flux (F60S) membrane. The BSA rejection of PVDF membrane was only 69.2%, which was lower than F60S membrane [2]. Now, we are trying to improve the PVDF hollow fiber hemodialysis membranes separation and other properties by optimizing membrane morphology and structure. The dialysis performance of PVDF dialyzer was also evaluated in this study.

## 2. Materials and Methods

### 2.1. Materials

The Polyvinylidene fluoride (SOLEF 1010) was purchased from Solvay So lexis Company (Lyon, France). Polyethylene glycol was purchased from Sigma-Aldrich Trading Co., Ltd. (Shanghai, China). Industrial grade N, N-dimethylacetamide (DMAc) was purchased from Samsung Company (Seoul, Korea). Lysozyme (LZM), Albumin from bovine serum (BSA) and urea were purchased from Shanghai biomedical engineering technical service company (Shanghai, China). 

### 2.2. Preparation of PVDF Hollow Fiber Membranes

PVDF, Polyethylene glycol, N, N-dimethylacetamide (DMAc) and 1,4-diethylene dioxide were dissolved in a glass flask at 70 °C. The content of PVDF was about 22 wt %, PEG content was 18.8 wt % and 1, 4-diethylene dioxide was 3 wt %. The casting dopes were dropped into the container and air bubbles were eliminated. Subsequently, the PVDF membranes were prepared by the non-solvent-induced phase separation (NIPS) method using a tube-in orifice spinneret. The spinneret used had an inner diameter of 0.7 mm and an outer diameter of 1.4 mm. The external and internal coagulants were pure water mixed with DMAc. The bore flow rate was 4.5 mL/min and the uptake speed was 72 m/min.

### 2.3. Membranes Characterization

#### 2.3.1. Morphology, Max Pore Size and Porosity

The PVDF membranes morphology structure was studied using a scanning electron microscope (Hitachi S-4800, HITACHI, and Tokyo, Japan). The SEM micrographs of PVDF membranes are M-1, M-2, M-3 and M-4 with PEG molecular weights of 2, 4, 6 and 10 kDa, respectively. The SEM micrographs of PVDF membranes are M-14.8, M-16.8, M-3 and M-20.8 with PEG content 14.8, 16.8, 18.8 and 20.8 wt %, respectively. M-0 was the membrane, which was prepared by the previous study [2].

The membrane, which has been soaked in ethanol for about 15 min, was immersed in ethyl alcohol. The bubble point pressure, *P*, was reached when the first string of bubbles came from the walls of the membrane with nitrogen. 

The maximum pore size can be calculated according to Equation (1) [19]:

(1) $$r=\frac{0.06378}{2P}$$

where *r* is the pore radius (µm); *P* is bubble point pressure (MPa); and the ethanol surface tension is 22.3 mN/m.

**Figure 1:**
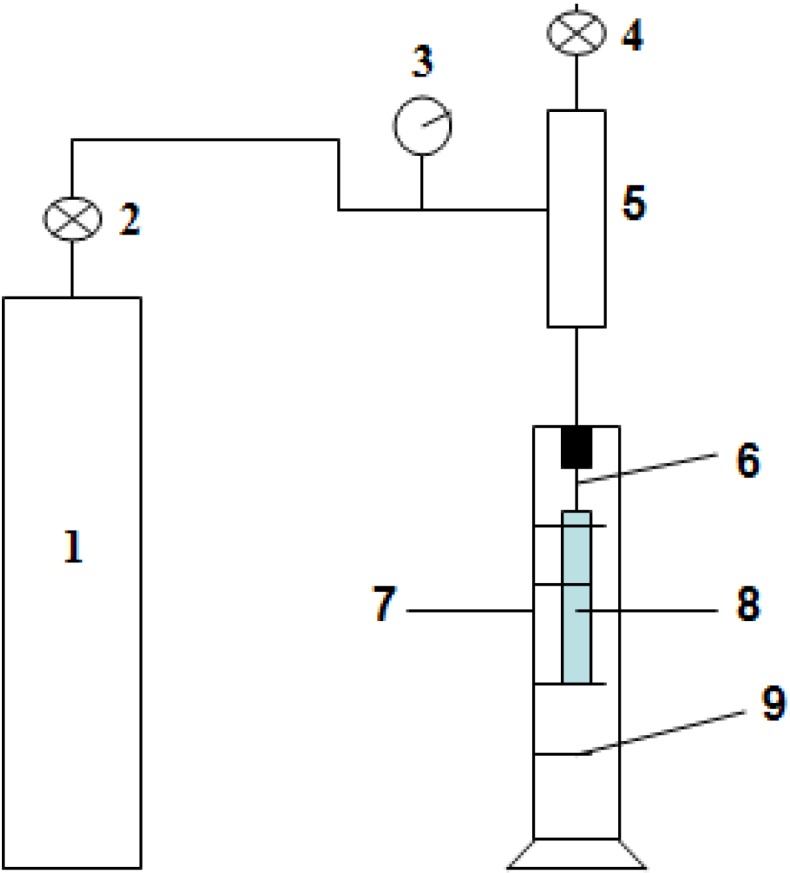
The apparatus for determining the Max pore size of the hollow fiber membranes [2] 1: nitrogen bottle; 2: regulator; 3: precise pressure gauge; 4: valve; 5: container; 6: syringe needles; 7: Transparent cylinder; 8: PVDF membrane sample to be tested; and 9: absolute ethyl alcohol.

The membrane porosity, *ε*, was defined as the volume of the pores divided by the total volume of the porous membrane. The membrane was soaked in ethanol for about 15 min, and than immersed in pure water.

The porosity was calculated using Equation (2):

(2) $$\varepsilon =\frac{\left(W_{w}-W_{d}\right)/\rho_{W}}{\left(W_{w}-W_{d}\right)/\rho_{W}+W_{d}/\rho_{p}}\times 100\%$$

where *ε* is the porosity of the membrane (%); *W_w_* is the mass of the wet membrane; *W_d_* is the mass of the dry membrane; *ρ_w_* is the density of water (1.0 g/cm^3^) and *ρ_p_* is the density of the membrane (1.78 g/cm^3^).

#### 2.3.2. Mechanical Properties, UF Flux of Pure Water and Rejection of BSA

Mechanical properties of the fabricated membranes were measured with an electronic single yarn strength tester (YG061 F/PC, Lanzhou Electron Instrument Co., Ltd., Lanzhou, China) at room temperature. The experiments were repeated five times and averaged.

Self-assembly widgets were made with 20 pieces of PVDF hollow fiber membranes by epoxy resin cast. The surface area of membranes was 25 cm^2^. The membranes were preloaded under 0.2 MPa for about 20 m. After adjusting the test temperature (25 °C), The UF flux was measured using the inlet pressure (0.102 MPa) and the outlet pressure (0.098 MPa). The transmembrane pressure (TMP) was 0.1 MPa. BSA rejection was measured using the same method as UF flux. 

The UF flux and BSA rejection of PVDF membranes were calculated according to our previous study [2]. 

Bursting pressure is a mechanical performance parameter of membranes. The membrane will be damaged when the pressure reaches bursting pressure. The value of bursting pressure was measured using the same equipment (Figure 1) as the Max pore size.

### 2.4. Dialysis Performance Test

#### 2.4.1. Selection of Standard Dialysis Solution

In this study, β_2_-MG and human serum albumin were replaced by Lysozyme and BSA. In this study, urea was chosen to characterize dialysis performance for removal of small molecules. In order to facilitate research, dialysis solutions were prepared by water, urea, LZM and BSA. The concentration of urea, LZM and BSA were 2000, 35 and 1000 mg/L, respectively.

#### 2.4.2. Dialysis Simulation

The standard dialysis solution comprised pure water, urea, lysozyme and bovine serum albumin. The flow rate of the simulated dialysis fluid was 200 mL/min. The concentrations of the simulated dialysis fluid were measured with UV-Vis spectrophotometer (TU-1810, Purkinje, Beijing, China). The dialysis time was about 4 h.

#### 2.4.3. Rejection of BSA and Clearance Rate of LZM and Urea

BSA rejection as well as Urea and LZM passage was measured using the same method as UF flux. After pre-flushing the membranes with the solute for about 30 minutes at a temperature of 25 °C, The BSA rejection as well as Urea and LZM passage of membranes were measured at the inlet pressure (0.100 MPa) and outlet pressure (0.0600 MPa).

The rejection of BSA (R) was calculated by the following Equation (5):

(3) $$R=\frac{Cp}{Cf}$$

where *C_p_* and *C_f_* (mg∙L^−1^) are BSA concentrations of after dialysis and Pre-dialysis solution, respectively.

The clearance rate of LZM and urea was calculated by the following Equation (6):

(4) $$R=1-\frac{Cp}{Cf}$$

where *C_p_* and *C_f_* (mg∙L^−1^) are LZM and urea concentrations of after dialysis and Pre-dialysis solution, respectively; the concentration was determined by UV-V is spectrophotometer.

## 3. Results and Discussion

### 3.1. Morphology and Structure of PVDF Membranes

#### 3.1.1. Morphology and Structure of Different PEG Molecular Weight

**Figure 2:**
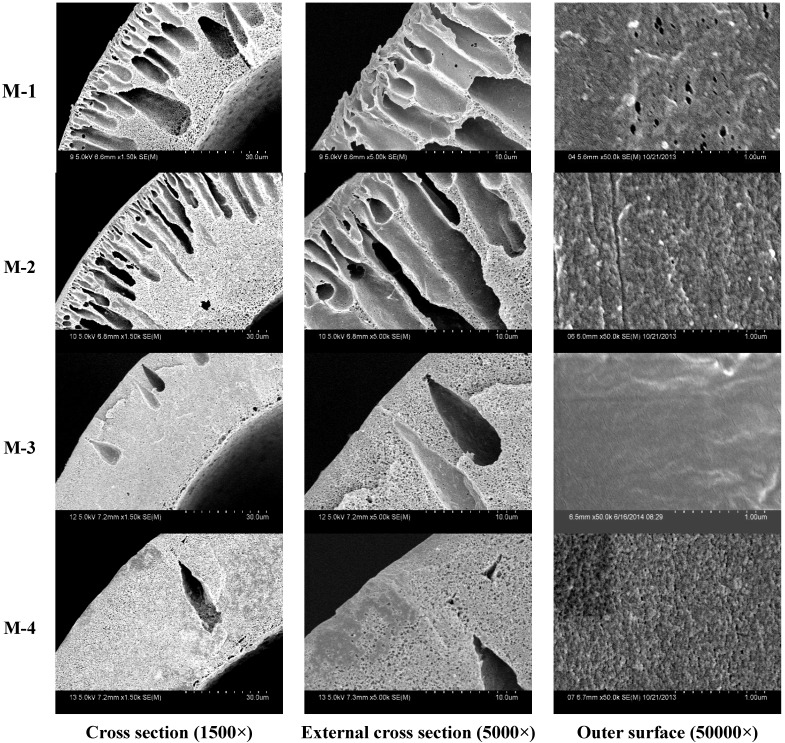
The SEM morphologies of different PVDF membranes, the labels M-1, M-2, M-3 and M-4 are membranes with PEG molecular weights 2, 4, 6 and 10 kDa, respectively.

The PVDF hollow fiber membranes SEM morphologies are shown in Figure 2. In this study, the PVDF membranes were prepared by NIPS method. The membrane matrix is PVDF. Polyethylene glycol and 1, 4-diethylene dioxide are modifiers to enhance membrane hydrophilicity. There was typical asymmetric structure in PVDF membranes. The structure was made of a skin layer, an intermediate layer with finger-like structure, and a bottom layer with fully developed macrospores. Finger-like structures dominate the cross section in M-1 and M-2 membranes. The sponge-structure in M-3 membrane becomes more and more obvious when compared to other membranes. There are some defective structures in M-4 membrane. The altered viscosity influences membrane structures. When PEG molecular weight is not very high (2–4 kDa), the viscosities of doping solutions changed slightly (Table 1). In that case, addition of PEG polymers has little influence on membrane structures. As PEG molecular weight is high (6 kDa), the asymmetric structure becomes more distinct. The casting solution viscosity becomes bigger and bigger with PEG molecular weight increasing when PVDF concentration is under certain preconditions, which can decrease the formation of macro voids [20]. The higher weight PEG can effectively influence the diffusion speed of casting solution, which can increase the formation of thick dense asymmetric layer. That can be explained by the diffusivity of additives and the solvent. Generally, the solvent is much faster than higher molecular weight PEG. However, when PEG molecular weight continues to increase (10 kDa), the casting solution stability is deteriorated and some defective pores appear in the PVDF membrane.

**Table 1:** Selected performances of different PVDF membranes; the labels M-1, M-2, M-3 and M-4 are membranes with PEG molecular weights 2, 4, 6 and 10 kDa, respectively.

Membrane Label	Porosity (%)	Bursting pressure (MPa)	Viscosity (mPa∙s)
M-1	88.9	0.395	3136
M-2	87.3	0.375	3421
M-3	85.1	0.625	3976
M-4	87.8	0.465	7352

**Figure 3:**
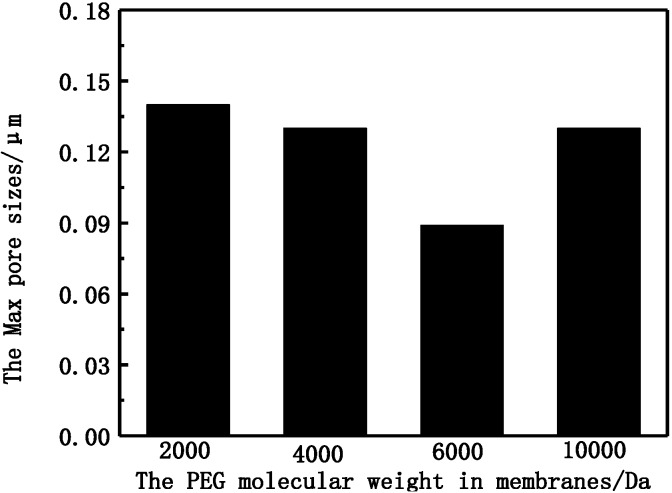
The max pore size of PVDF membranes with different PEG molecular weight

From SEM morphologies of different PVDF membranes, it can be seen that the outer surface of the M-3 membrane is much denser, and the max pore size cannot be observed clearly while the outer surface of other membranes is rough and porous. The formation of pores becomes suppressed and smaller in the M-3 membrane. The diminishing of finger-like pores can increase the separation properties of PVDF membranes [21]. The max pore size of different membranes is shown in Figure 3.

#### 3.1.2. Morphology and Structure of Different PEG Content

**Figure 4:**
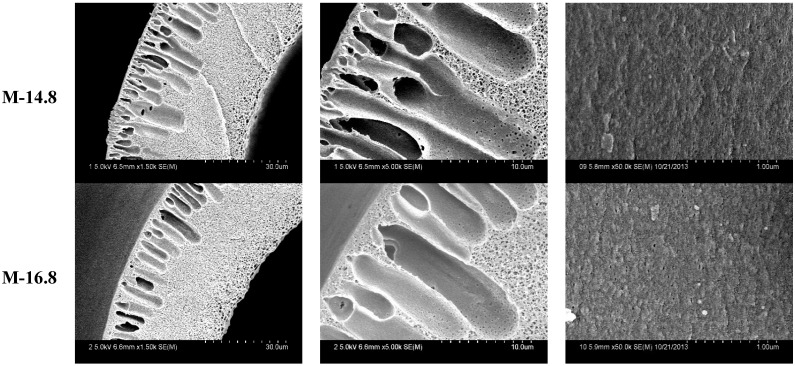

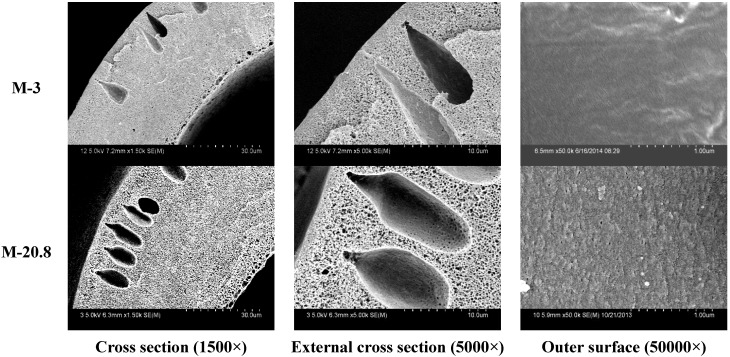
The SEM morphologies of different PVDF membranes, the labels M-14.8, M-16.8, M-3 and M-20.8 are membranes with PEG content 14.8, 16.8, 18.8 and 20.8 wt %.

The PVDF hollow fiber membranes SEM morphologies with different PEG content are shown in Figure 4. There was typical asymmetric structure in different PVDF membranes. The structure was made of a skin layer; an intermediate layer with finger-like structure; and a bottom layer with fully developed macrospores [22]. Finger-like structure dominates the cross section in M-14.8 and M-16.8 membranes. The sponge-structure in M-3 membrane becomes more and more obvious when compared to other membranes that have more finger-like structure. There are some defective structures in M-20.8 membrane. That can be explained by the solvent and non-solvent diffusion rate.

**Figure 5:**
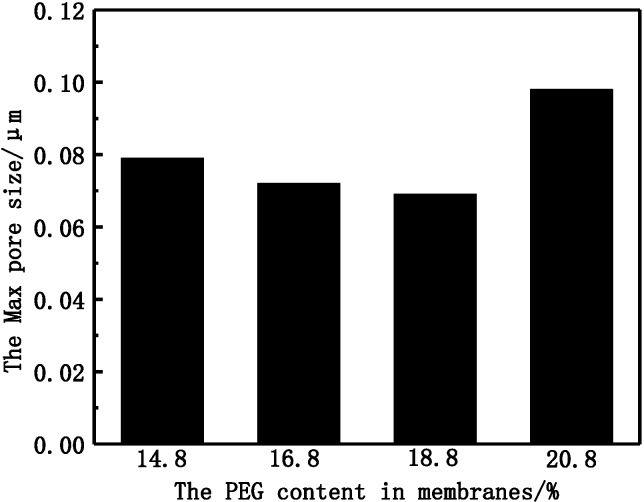
The max pore size of PVDF membranes with different PEG content.

The outer surfaces of different PVDF membranes are shown in Figure 5. With PEG content ranged from 14.8 wt % to 18.8 wt %, the outer surface changed from porous to dense. As PEG content reached 20.8 wt %, the surface becomes porous again.

### 3.2. Mechanical and Separation Performance of PVDF Membranes

#### 3.2.1. Mechanical and Separation Performance of PVDF Membranes with Different PEG Molecular Weight

**Figure 6:**
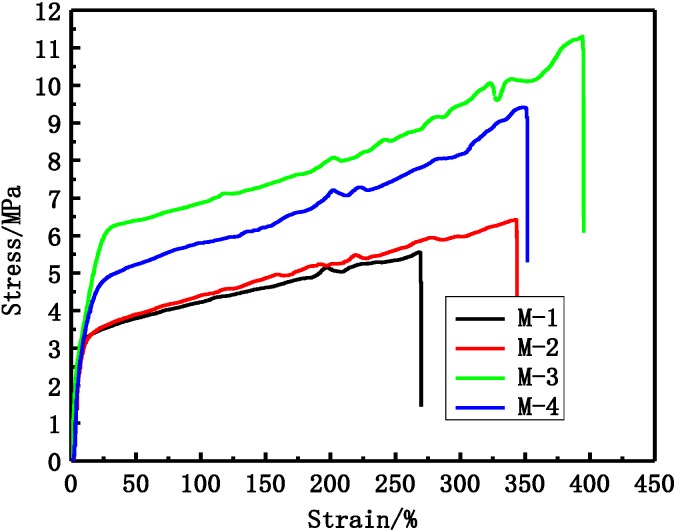
The stress-strain curves of different PVDF membranes, the labels M-1, M-2, M-3 and M-4 are membranes with PEG molecular weights 2, 4, 6 and 10 kDa, respectively.

The tensile stress and bursting pressure at break increases with increasing PEG molecular weight at first, and then decreases, as shown in Table 1 and Figure 6. M-3 membrane has the best mechanical properties. The membrane tensile stress at break is 11.3 MPa and bursting pressure at break is 0.625 MPa. This can be explained by two reasons: (1) the cross-sectional structure can affect the membrane mechanical performance. From morphologies (Figure 2), it can be seen that the finger-like structure becomes less and less with increasing PEG molecular weigh, which can increase the tensile stress. With PEG molecular weight continuing to increase, the casting solution stability is deteriorated and the membrane mechanical performance begins to decrease; (2) the porosity can also affect the mechanical performance. From Table 1, the porosity decreases from 88.9% to 85.1%, and then increases to 87.8%. The higher porosity membrane has lower mechanical performance.

The BSA rejection rate of different PVDF membranes is shown in Table 2. It is observed that the M-3 membrane exhibits the highest BSA rejection (82.3%) while M-4 shows the lowest. These results can be illustrated by different outer surfaces of dialysis membranes. The outer surface becomes denser with PEG molecular weight increasing at first, and then becomes looser (as shown in Figure 2). Recently, high molecular weight additives have become more and more popular in the preparation of blood dialysis membranes, which can form dense layers easily. Meanwhile, dense structure can increase BSA rejection. This result is consistent with the study of Yuan *et al*. [23]. The study showed that the surface roughness of membranes was effectively reduced with molecular weight of PEG increasing. With the PEG molecular weight continuing to increase, the casting solution stability is deteriorated and easily forms lager pore sizes, which decreases the separation performance of membranes.

**Table 2:** Separation performance and water contact angle of PVDF membranes with different PEG molecular weight.

Membrane label	UF flux of pure water (L∙h^−1^∙m^−2^)	Rejection of BSA (%)	Water contact angle (°)
M-1	45.2	4.4	57 ± 3
M-2	35.4	40.9	54 ± 2
M-3	108.2	82.3	52 ± 2
M-4	124.8	8.8	42 ± 2

As shown in Table 2, UF flux of pure water increases when PEG molecular weight increases. From the study of Jung *et al.* [24], it is well known that the solubility of additives decreases when molecular weight increases. On the one hand, low molecular weight additives can easily be washed out together with the solvent from membranes. On the other hand, the higher molecular weight additives are often left in the membranes. There are more additives that stay in the membranes. PEG is an uncharged polymer with hydrophilicity, which can increase the hydrophilicity of PVDF membranes.

#### 3.2.2. Mechanical and Separation Performance of PVDF Membranes with Different PEG Content

**Figure 7:**
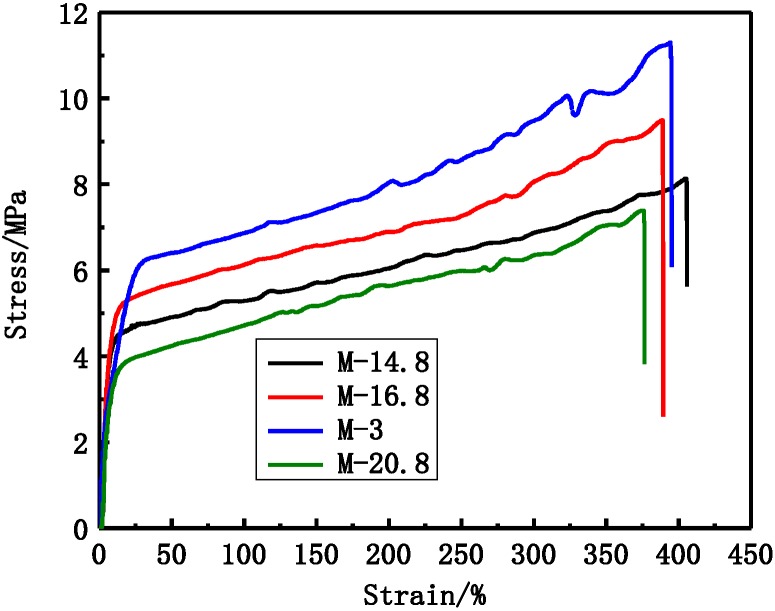
The stress-strain curves of different PVDF membranes; the labels M-14.8, M-16.8, M-3, and M-20.8 are membranes with PEG content: 14.8, 16.8, 18.8 and 20.8 wt %, respectively.

As shown in Table 3 and Figure 7, the tensile stress increases from 7.4 MPa to 11.3 MPa and the porosity decreases from 88.8% to 85.1% at first, and then the tensile stress decreases from 11.3 MPa to 8.1 MPa and the porosity increases from 85.1% to 87.5%. The results also indicate that the bursting pressure increases with membrane porosity decreasing. Therefore, both the casting solution composition and membrane porosity can affect the tensile stress at break. The M-3 membrane has better mechanical properties than the other PVDF hollow fiber membranes.

**Table 3:** Selected performance of different PVDF membranes; the labels M-14.8, M-16.8, M-3, and M-20.8 are membranes with PEG content: 14.8, 16.8, 18.8 and 20.8 wt %, respectively.

Membrane label	Porosity (%)	Bursting pressure (MPa)	Viscosity (mPa.s)
M-14.8	88.8	0.495	2736
M-16.8	87.3	0.605	3124
M-3	85.1	0.625	3976
M-20.8	87.5	0.510	2846

UF flux, BSA rejection and water contact angle results of PVDF membranes with different PEG content are shown in Table 4. BSA rejection increases from 60.6% to 82.3% as a result of denser surface structure, which makes the max pore size smaller (As shown in Figure 4). With PEG content continuing to increase, the BSA rejection decreases from 82.3% to 70.2%. That can be explained by the max pore size becoming larger again. The UF flux increases with increasing PEG content. From the study of Kim and Lee, it is well known that PEG content can effectively improve the water permeability of membranes [25].

**Table 4:** Separation performance and contact angle of PVDF membranes with different PEG content.

Membrane label	UF flux of pure water (L∙h^−1^∙m^−2^)	Rejection of BSA (%)	Water contact angle (°)
M-14.8	45.5	60.6	59 ± 3
M-16.8	65.4	66.2	56 ± 2
M-3	108.2	82.3	52 ± 2
M-20.8	106.6	70.2	43 ± 2

### 3.3. Contrast of PVDF and F60S Membranes

#### 3.3.1. Morphology 

**Figure 8:**
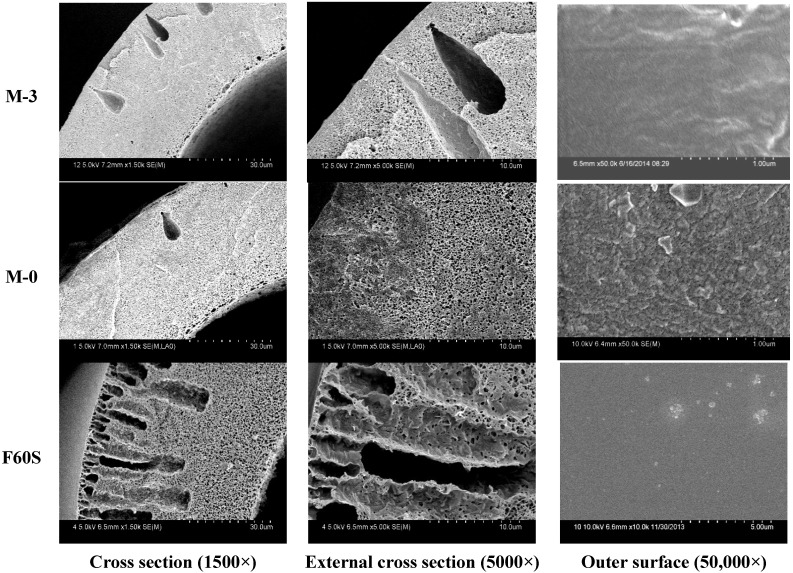
The PVDF and F60S membranes SEM morphologies; M-0 is the PVDF membrane that was prepared in a previous study [2].

The SEM micrographs of PVDF and Fresenius F60S membrane are shown in Figure 8. The Fresenius F60S membrane exhibits more finger-like pores while there are few finger-like structures in M-3 and M-0 membranes. The outer surface of M-0 is rougher and more porous than M-3 and F60S membranes.

#### 3.3.2. Mechanical and Separation Properties

**Figure 9:**
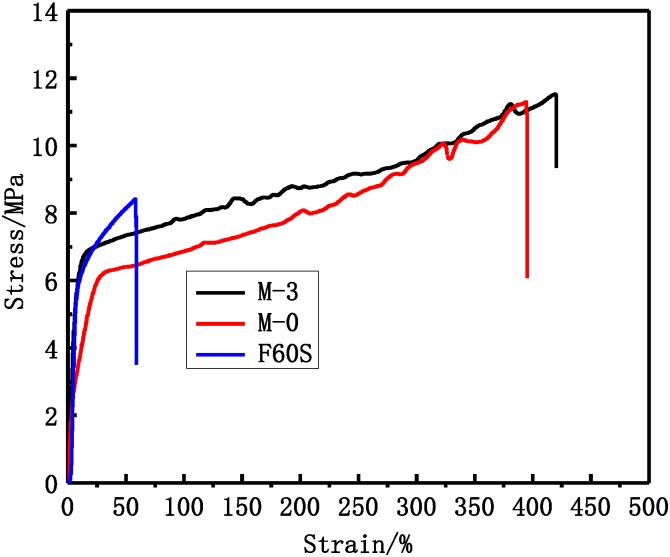
The PVDF and F60S membranes stress-strain curve, M-0 is the PVDF membrane that was prepared in a previous study [2].

From Figure 9 the results show that the stretching strength of different PVDF membranes is much stronger than F60S membrane. At the same time, the stretching strain of M-3 and M-0 membranes is about 400%, which is much higher than F60S membrane. The tensile stress at break of PVDF membranes was about 11 MP and much higher than that of F60S membrane (7.9 MPa). This can be explained by different materials and different morphology structures (Such as Figure 9). UF flux and BSA rejection of different membranes are shown in Table 5. UF flux and BSA rejection of M-3 membrane are 108.2 L∙h^−1^∙m^−2^ and 82.3%, respectively, which are higher than M-0 (98.7 L∙h^−1^∙m^−2^ and 69.2%) and F60S membranes. The F60S membrane UF flux is 78.6 L∙h^−1^∙m^−2^ and BSA rejection is 78.2%. Compared with F60S membrane; the M-3 membrane has better mechanical and separation properties.

**Table 5:** Bursting pressure, Rejection of BSA and UF flux of pure water of different membranes.

Membrane label	Bursting pressure (MPa)	Rejection of BSA (%)	UF flux of pure water(L∙h^−1^m^−2^)
M-3	0.625	82.3	108.2
M-0	0.645	69.2	98.7
F60S	0.475	78.2	78.6

### 3.4. Research of PVDF Dialyzer

#### 3.4.1. UF Coefficient of PVDF Dialyzer

**Figure 10:**
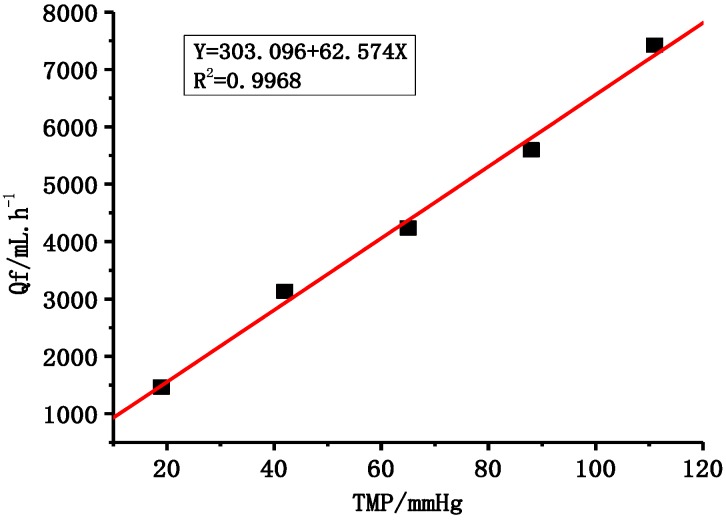
The UF coefficient of PVDF dialyzer.

PVDF dialyzers were prepared using M-3 membrane. Membrane permeability to water is generally expressed by UF coefficient. From Figure 10, it can be seen that the PVDF dialyzer UF coefficient reaches 62.6 mL/h/mmHg.

#### 3.4.2. Dialysis Performance of PVDF Dialyzer

From Table 6, it can be seen that the dialyzers with different membrane area have different dialysis performance to solutes under the same dialysis conditions. The rejection of bovine serum albumin is similar that can reach 80% while the clearance rate to lysozyme and urea is different. The clearance rate to Lysozyme is 63%, 66.8% and 57.4%, respectively. The clearance rate to urea is 78.8%, 87.7% and 82.5%. The PVDF dialyzer with membrane area 0.69 m^2^ has highest clearance rate to LZM and urea. This is because the exchange rate between dialysis and simulation fluid increases with increasing membrane area. The exchange rate can be limited with membrane area continuing to increase. That can be due to a certain area of the dialysis shell.

**Table 6:** Dialysis performance of different dialyzer area.

Area (m^2^)	Concentration of BSA /mg∙L^−1^			Concentration of LZM /mg∙L^−1^			Concentration of Urea /mg∙L^−1^		
	Pre dialysis	After dialysis	Rejection (%)	Pre dialysis	After dialysis	Clearance (%)	Pre dialysis	After dialysis	Clearance (%)
0.43	934	767	82.1	36.2	13.4	63.0	2080	442	78.8
0.69	934	770	83.1	36.2	12.4	66.8	2080	283	87.7
0.95	934	781	83.6	36.2	11.8	57.4	2080	364	82.5

#### 3.4.3. Dialysis Performance of Different Simulation Fluid Flow Rate

Table 7 shows that BSA rejection is almost the same under three flow rates of simulation fluid. The retention rate of BSA is 82.0%, 81.6% and 83.1%, respectively, which indicates that increasing simulation fluid flow rates has little effect on the retention of BSA. The clearance rate of lysozyme increases from 61.0% to 66.8% with the simulation fluid flow rate increasing from 100 mL/min to 200 mL/min. The clearance rate of Urea is also different under three simulation fluid flow rates; when the flow rate increased from 100mL/min to 200 mL/min, the clearance rate increased from 84.3% to 87.7%. From the results, it can be seen that increasing simulation fluid flow rate can improve the clearance rate of lysozyme and Urea.

**Table 7:** Dialysis performance at different simulation fluid flow rates.

Flow rate (mL/min)	Concentration of BSA /mg∙L^−1^			Concentration of LZM /mg∙L^−1^			Concentration of Urea /mg∙L^−1^		
	Pre dialysis	After dialysis	Rejection (%)	Pre dialysis	After dialysis	Clearance (%)	Pre dialysis	After dialysis	Clearance (%)
100	934	765	82.0	36.2	14.1	61.0	2080	326	84.3
150	934	762	81.6	36.2	13.5	62.7	2080	320	84.6
200	934	770	83.1	36.2	12.4	66.8	2080	283	87.7

## 4. Conclusions

The membrane morphology structure can affect the mechanical and separation performance of PVDF membranes. The M-3 membrane with PEG molecular weight of 6 kDa and content 18.8 wt % has the best mechanical and separation properties when compared to other PVDF membranes with respect to optimized membrane morphology and structure. The PVDF hemodialysis membrane has better mechanical and separation properties compared to medical F60S membrane. The PVDF membrane tensile stress at break, tensile elongation, and bursting pressure were 11.3 MPa, 395% and 0.625MPa, respectively, while F60S membrane were 7.9 MPa, 59% and 0.475 MPa, respectively. UF flux and BSA rejection of M-3 membrane are 108.2 L∙h^−1^∙m^−2^ and 82.3%, which are higher than M-0 (98.7 L∙h^−1^∙m^−2^ and 69.2%) and F60S membranes. The F60S membrane UF flux is 78.6 L∙h^−1^∙m^−2^ and BSA rejection is 78.2%. Dialyzer membrane area and simulated fluid flow rate affect dialysis performance. The clearance rate of urea and LZM were improved by increasing the membrane area and fluid flow rate, while this had little influence on the rejection of BSA. The high-flux PVDF dialyzer, with 62.6 mL/h/mmHg (UF coefficient), had better clearance rates for LZM and urea. The clearance rate of LZM was 66.8% and urea was 87.7% in for a 4 h dialysis process.
